# Transgenerational epigenetic reprogramming of early embryos: a mechanistic model

**DOI:** 10.1093/eep/dvaa009

**Published:** 2020-07-18

**Authors:** Corrado Spadafora

**Affiliations:** Institute of Translational Pharmacology, National Research Council (CNR), 100 Via del Fosso del Cavaliere, 00133 Rome, Italy

**Keywords:** epigenetic inheritance, sperm cells, extracellular vesicles, early embryos, chromatin remodeling, sperm RNA, Waddington

## Abstract

The notion that epigenetic information can be transmitted across generations is supported by mounting waves of data, but the underlying mechanisms remain elusive. Here, a model is proposed which combines different lines of experimental evidence. First, it has been shown that somatic tissues exposed to stressing stimuli release circulating RNA-containing extracellular vesicles; second, epididymal spermatozoa can take up, internalize and deliver the RNA-containing extracellular vesicles to oocytes at fertilization; third, early embryos can process RNA-based information. These elements constitute the building blocks upon which the model is built. The model proposes that a continuous stream of epigenetic information flows from parental somatic tissues to the developing embryos. The flow can cross the Weismann barrier, is mediated by circulating vesicles and epididymal spermatozoa, and has the potential to generate epigenetic traits that are then stably acquired in the offspring. In a broader perspective, it emerges that a natural ‘assembly line’ operates continuously, aiming at passing the parental epigenetic blueprint in growing embryos.

## Multifactorial Epigenetic Entanglement

Living organisms are endowed with an amazing potential to generate phenotypic novelties, not necessarily caused only by random genetic mutations but also driven by epigenetic processes triggered in response to a variety of environmental stimuli [1, 2 and references herein]. In mammals, these epigenetic variations can be transmitted via gametes across one or multiple generations, with variable efficacy depending on the nature and intensity of the stressing stimulus and, possibly, on other as yet unidentified parameters.

More specifically, an increasing body of evidence shows that the exposure of cells and organisms to various stimuli, including environmental toxics [3, 4], dietary intake [5], substance abuse (alcohol, nicotine, drugs) [6] and even psychological stressors [7] and traumatic experience [8], can all trigger variable epigenetic modifications responsible for an ample spectrum of phenotypic effects, including adverse health consequences, transmissible to the progeny (reviewed in Ref. [9, 10]). Thus, a subtle flow of extrachromosomal information [11] connects the environment with endogenous epigenetic mechanisms highly sensitive to the external conditions. Together, these data have inspired a vision of the genome as an interactive entity endowed with information-processing networks at work in living organisms [12].

Classical epigenetic variations are incorporated as genome-shaping marks, such as DNA methylation [13] and histone modifications [14, 15]. In addition, epigenetic variations can also be mediated via modulation of the expression of regulatory small [16] and long [17] non-coding RNAs. These key processes profoundly impact the organization of the nuclear architecture, inducing a global reprogramming of the expression profile of the cell [18, 19].

Epigenetic alterations can be transmitted to the progeny in one or across multiple generations. It is important to distinguish between ‘intergenerational’ effects, when the environmental stimulus is germline-independent and directly affects gestating embryos, and ‘transgenerational’ effects, when the latter are inherited from one generation to the next through the germline, even without exposure of the next generation to the original triggering stimulus [2]. That distinction is relevant because only transgenerational inheritance implies an active role for gametes. To avoid the so-called maternal ambiguity—which makes it impossible to discriminate whether a novel change appearing in the offspring was maternally transmitted via the epigenetic state of the oocyte, or directly acquired in the embryo during gestation—many research efforts have focused on paternal effects; sperm cells have thus become the object of intense investigation as transgenerational vectors of epigenetic information [20, 21]. It is now well established that, at fertilization, spermatozoa, concomitant with the paternal genome, also deliver to the oocyte ‘quanta’ of RNA-based epigenetic information of heterogeneous composition, which—amazingly—can reflect the paternal health conditions and even his broader life experiences (reviewed in Ref. [20, 22, 23]).

Converging evidence suggests that extrachromosomal information provides the molecular tools that trigger the transgenerational emergence of altered traits in the developing progeny. The molecular mechanism through which such variations are acquired in the embryos, however, remains elusive. Based on published experimental data, what follows is a hypothetical mechanistic model illustrating the molecular machinery that enables the decoding of the delivered extrachromosomal information in the permissive context offered by early embryos, and hence the emergence of altered phenotypic features.

## Structure of the Proposed Model

The model proposes a stepwise process in which novel epigenetic information, under the form of small regulatory RNAs, is sequentially generated in somatic tissues, packaged in extracellular vesicles (ECVs), and delivered to early embryos, where they ultimately drive a global reprogramming of genome expression. The model is, therefore, built on three sequential phases, as sketched out below.

Phase 1: RNA-based information, predominantly constituted by small regulatory tRNA-derived small RNAs and miRNAs, is in tissues in response to stressing environmental stimuli and is packed in ECVs, that can then be released from tissues in different districts of the body to the blood stream.

Phase 2: RNA-containing ECVs circulating in the blood stream are eventually taken up by epididymal spermatozoa and internalized in nuclei. The uptake of this extracellular RNA generates an RNA ‘storage’ in the sperm. As said above, these RNAs have a heterogeneous composition and include a large population of small regulatory RNAs that vary with both the original cellular sources and the nature of the stressing stimulus. For example, sperm cells from obese animals contain elements of specific coregulatory networks that include RNAs, chromatin modifiers, as well as a large number of sequences associated with obesity-related inflammation, adipogenesis and cellular stress [24].

Phase 3: At fertilization spermatozoa deliver to oocytes their RNA cargo, which propagates further in zygotic pronuclei and in two- and four-cell embryos, as indicated by the evidence that sperm RNAs persist in early embryos [25]. The remodeling of the transcription landscape driven by the newly delivered regulatory RNAs starts soon after fertilization, in both the maternal and paternal pronuclei, and proceeds in one- and two-cell embryonic nuclei, which are in a ‘permissive’ condition at these stages. Ultimately, this process reprograms the expression profile in the embryo, thereby favoring the emergence of phenotypic alterations.

## Regulatory RNA-Containing ECVs Are Released in the Bloodstream

ECVs, including exosomes, are heterogeneous membrane-bound particles released from tissues and can carry heterogeneous DNA, RNA, proteins and metabolites (extensively reviewed in Ref. [26]) delivered from and exchanged both between different cell types [27] and across species [28]. ECVs play crucial roles as horizontal vectors in many physiological functions, including homeostasis maintenance [26], and as diverse pathological processes as cancer, cardiometabolic, neurologic and infectious diseases [29]. The RNA cargo of ECVs can comprise mRNAs, miRNAs, tRNA-derived small RNAs, long non-coding RNA and circular RNA. Interestingly, comparative studies show that the composition of the RNAs contained in secreted ECVs can differ from that of their originating cells, suggesting that these RNAs are the product of an active sorting process. The evidence that ECVs contain specific RNA profiles suggest that RNAs are not passively packed into vesicles but undergo a ‘seaving’ process. Efforts to characterize the molecular components of the process have identified both the presence of specific motifs in selected miRNA populations, and specific RNA-binding proteins implicated in RNA loading into ECVs [30, 31]. As a result, selected RNA populations are eventually preferentially enriched in ECVs compared to the original RNA profile present in parental cells. Importantly, the ECV RNA cargo is heavily influenced by the stressing conditions to which the delivering cells were exposed ([32], reviewed in Ref. [33, 34]). From these data, ECVs act as vectors of a specific, variable, RNA populations that arise from the combination of: (i) different originating tissues, (ii) selective sorting from cell donors and (iii) different stressing stimuli targeting the original cell sources.

Remarkably, ECVs released from tumor cells contain high levels of transcripts from LINE-1 and Alu retrotransposons and are also endowed with reverse transcriptase (RT) activity [35]. Strikingly, it has been shown that ECVs released from cancer cells can vehiculate specific signals and cancer traits that affect the function, phenotype and fate not only of the neighboring cells but also of distant cells, which they can reach through biological fluids [36, 37]. To summarize, ECVs with variable RNA loads are generated in and released from different tissues in the blood stream, and can convey their information to different districts of the organism, with the potential to induce epigenetic changes and/or reprogram the expression profiles of the targeted cells.

## Crossing the Weismann Barrier: Epididymal Spermatozoa as Collectors of Circulating ECVs—The Sperm ‘RNA Code’

Circulating ECVs reach the epididymis, are taken up in mature spermatozoa and internalized in their sperm head. It has been demonstrated that the ECV-delivered RNAs contribute a large proportion of the bulk RNA in spermatozoa [38]. Independent lines of evidence show that epididymal spermatozoa can spontaneously take up exogenous DNA and RNA molecules and internalize them in the nuclear chromatin [39–41]. More recently, it has been shown that epididymal spermatozoa are also spontaneously permeable to whole exosomes [42]. In contrast, ejaculated spermatozoa are unable to bind foreign molecules as long as they are immersed in the seminal fluid, but their permeability is restored after thorough washes that remove even trace amounts of seminal fluid (reviewed in Ref. [39]). The ejaculated seminal fluid actually contains factors abrogating the sperm permeability. These factors provide a natural ‘plug’, and hence constitute a barrier against undesired intrusions in ejaculated spermatozoa [39], a condition under which the highly reactive sperm cells might otherwise come in contact with spurious exogenous nucleic acid molecules, which could potentially compromise the genetic identity of the embryos. Indeed, converging conclusions from *in vitro* fertilization assays in mice, artificial insemination in large farm animals and external fertilization in fish and amphibia, indicate that seminal fluid-free spermatozoa are permeable to exogenous RNA and DNA molecules which they carry through and deliver to oocytes at fertilization. The foreign molecules propagate as non-integrated structures in developing embryos and are eventually mosaic-inherited, with variable efficacy, in tissues of born animals, where they are expressed as novel traits ([41, 43, 44], reviewed in Ref. [45]). This process, called sperm-mediated gene transfer, reveals the ability of mature sperm cells to take up and deliver foreign nucleic acids as a common feature in virtually all animal species, from echinoids to mammals, including humans [45]. Thus, epididymal spermatozoa can behave as collectors and vectors not only of their own genome but also of extrachromosomal DNA and RNA molecules and of circulating ECVs.

The interaction between epididymal spermatozoa and foreign nucleic acid molecules can spontaneously occur in nature. It has been recently shown that RNA can be transferred from neighboring epididymal tissue to spermatozoa, mediated by a special class of ECVs called epididymosomes [46–48]. In past work, our group engineered human melanoma cells to express an EGFP-encoding plasmid then inoculated them subcutaneously into nude mice; we found that the melanoma-derived EGFP RNA-containing ECVs were released in the mice bloodstream, reached the epididymis and eventually released their EGFP-encoding RNA in spermatozoa [49]. These data expand the concept of soma-to-germline transmission and show that RNA-based information does not only reach epididymal spermatozoa from the closely located epididymis [46–48], but can actually be transferred from distant somatic tissues [50]. Thus, somatic ECVs can actually cross the Weismann barrier [51] and interact with fertile male gametes. Consistent with this view, it is now accepted that most of the RNA stored in spermatozoa is not a product of transcription during spermatogenesis, but rather derives from an epididymosome-mediated soma-to-spermatozoa acquisition process that later unfolds during the epididymal maturation of sperm cells [47, 48].

Molecular studies show that spermatozoal RNA comprises an expanding repertoire of RNA classes with regulatory functions, including primarily tRNA fragments [52–54], representing the most abundant class of RNA in mature spermatozoa; miRNAs [46, 47]; rRNA-derived small RNAs [55] and long non-coding RNAs [56]. An increasing body of evidence confirms that environmental factors, such as dietary intake, exposure to toxics and even mental stress, impact on the spermatozoal RNA populations and hence can influence the offspring’s phenotype ([57–59], reviewed in Ref. [60]). The finding that injection of sperm RNA in zygotes recapitulates the paternally acquired phenotypes in offsprings confirms that sperm RNA act as the transgenerational ‘carrier’ of environmentally induced information [8, 53, 54, 61, 62]. Recent data further reveal the crucial roles played by epigenetic marks that differentially decorate the sperm RNA fractions in transgenerational inheritance (reviewed in [53, 63]). A novel epigenetic landscape is beginning to emerge, based on the evidence that post-transcriptional modifications expand the information capacity of sperm RNA beyond their primary sequence. In sperm RNA, the combination of the primary sequence and their modifications, both of which are sensitive to the paternal environment, constitutes a complex ‘RNA code’ carrying multiple layers of environmentally modulated information ready to be processed by the ‘decoding machinery’ active in preimplantation embryos [63]. The next section describes a model through which the RNA-based information can be ‘decoded’ and converted into phenotypical traits in the earliest embryonic transitions.

## Decoding the Sperm ‘RNA Code’ and the Reprogramming of Early Embryos

At fertilization, spermatozoa deliver extrachromosomal information to oocytes in the form of pure RNA [64] or RNA-containing ECVs [42, 49, 65]. Through this pivotal function, sperm cells bridge the gap across generations and directly transfer parental somatic RNA-based information to the developing progeny; this process bypasses the systematic epigenetic resetting occurring in meiosis and early embryogenesis (reviewed in Ref. [66]): thus, a parental transcription blueprint is passed to the embryo soon after fertilization.

**Figure 1:**
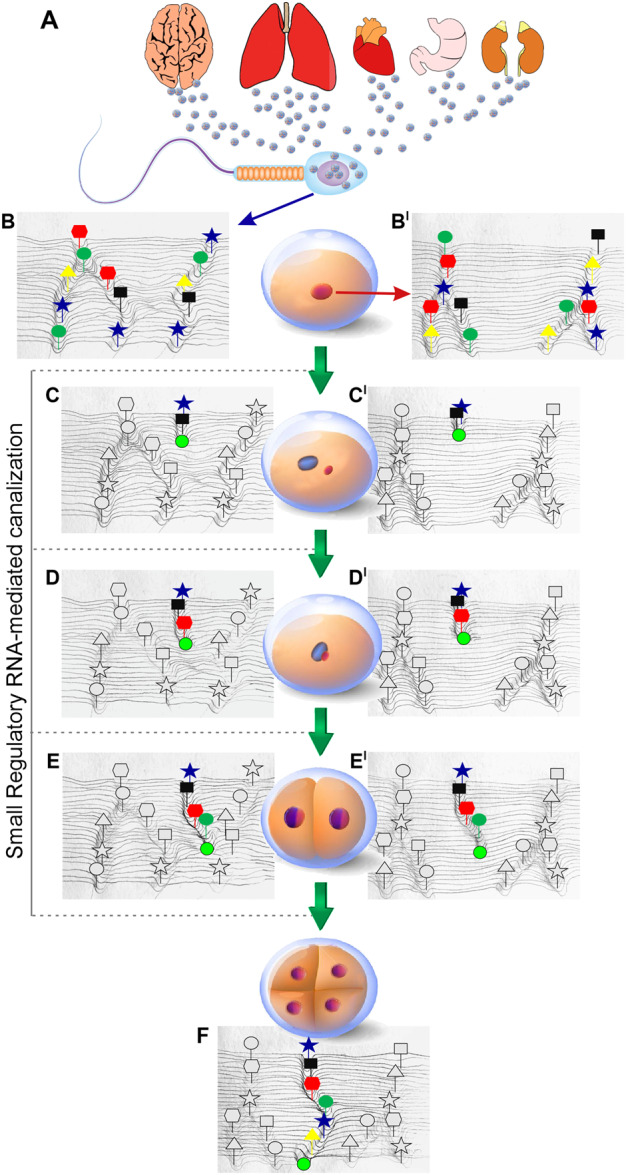
Schematic illustration, inspired by the Waddington’s model, representing the canalization process of the early embryonic epigenetic landscape. (**A**) RNA molecules are released from somatic tissues from various organs, packaged in extracellular vesicles (likely exosomes), released within the blood stream and eventually taken up by permeable epididymal spermatozoa. (**B** and **B**.) The epigenetic canalized landscapes in spermatozoa and oocytes, respectively (colored symbols). The canalization patterns are generated during gametogenesis. (**C** and **C’**) Canalization of the epigenetic landscape independently occurring in the sperm-derived (blue) and oocyte (red) zygotic pronuclei. The green ball rolling down from the hill top represents the initial trajectory of the progressing epigenetic canalization pathway driven by sperm-delivered regulatory RNAs. Targeted retrotransposal insertions (colored shapes) follow the progressing groove. (**D** and **D’**) Symbols are as in C, C’, with canalization progressing in the zygotic nucleus where the two parental genomes have not yet fused. (**E** and **E’**) Canalization in the nuclei of the two-cell stage embryo, where the two genomes are still not fused (blue/red halves). (**F**) The complete canalization pathway in the nuclei of four-cell embryos. Paternal and maternal genomes are now fused and the landscape progresses in a new single pathway. The deeper groove with colored symbols represents the functionally active path, while the grooves with gray symbols are not in use.

Figure 1 is inspired by the landscape concept originally proposed by Waddington [67], and represents the hypothetical unfolding of the canalization process driven by sperm-delivered regulatory RNAs in early embryogenesis. The model builds upon the evidence summarized above that RNA-containing ECVs are released from organs and tissues in the blood stream, reach the epididymis and therein are taken up by epididymal spermatozoa (A). The next steps take into account the notion that the epigenetic landscapes of sperm and oocyte (represented in panels B and B’, respectively, as the canalized pathways containing colored symbols) are set during gametogenesis, but the RNA cargo (represented by the green ball in panels C and C’) delivered from spermatozoa can reshape them at fertilization. Actually, the released RNA can trigger similar, yet independent, canalization processes in both paternal (C panel) and maternal (C’ panel) pronuclei. Spermatozoa harbor chromatin domains where histones are not replaced by protamines and the nucleosomal structure is preserved [68, 69]; it is reasonable to hypothesize that those domains represent compartments where the reshaping processes preferentially occur. Interestingly, sperm nucleohistone domains typically contain gene promoters and regulatory sequences in an ‘active’ chromatin conformation, with future developmental relevance [70–72]. The canalization continues in the nuclei of zygotes (D, D’) and two-cell embryos (E, E’), in which paternal and maternal genomes are still separated though being contained within the same nucleus [73, 74], and is complete at the four-cell stage, when the two genomes become fused (panel F). Concomitant with this, the canalizations that pre-existed in gametes (colored symbols in panels B and B’) are functionally inactivated (gray symbols, panels C–E, C’–E’, F).

These early chromatin remodeling events are central to the model. Recent findings show that basic chromatin structures, such as lamina-associated domains (LADs), i.e. large DNA-lamina interacting regions that contribute to the spatial distribution of chromosomes [75], are established *de novo* soon after fertilization [76]. At first LADs are independently organized in each of the two parental genomes, yet they come to converge after the eight-cell stage and thereafter maintain a flexible structure, as their spatial organization will be susceptible to variations during differentiation, development, senescence and cancer (reviewed in Ref. [77]). Interestingly, 3D nuclear structures within sperm chromatin, equivalent to those found in somatic cells, have been mapped; specific domains have been identified and proposed to serve as continuous landmarks that may facilitate the reconstitution of chromatin after fertilization [78].

Another fundamental contribution towards forming novel genomic circuits is provided by targeted retrotransposal insertions, represented by colored symbols placed on the wake of the progressing green ball along the newly ‘canalized pathways’ (panels C–E, C’–E’, F). The insertions involve LINE-1, HERV and Alu/SINEs, members of large retrotransposon families that use a ‘copy-and-paste’ mechanism for their amplification and mobilization: briefly, full-length genomic elements are first transcribed into RNA, followed by reverse-transcription of the RNA in cDNA copies that finally integrate stably in the host genome [79]. Germline insertions are then inherited across generations. These insertions have a dual function: (i) they stabilize the newly canalized pathways, by ‘nailing’ the regulatory circuits into the epigenetic landscape and (ii) they provide regulatory sequences, i.e. splicing sites, promoters, enhancers, insulators and binding sites for polymerases, hormones, transcription factors and so on [80, 81]. Integrations can only occur in portions of the host genome. Indeed, earlier experiments pinpointed nuclease-sensitive, i.e. structurally ‘accessible’ chromatin domains in sperm nuclei, predominantly constituted by retrotransposon sequences enriched in transcription factor-binding sites [82]. These domains conceivably represent preferential target sites for retrotransposon insertions.

Recent data show that families of repetitive elements are tightly associated with the expression of particular classes of genes during embryo development and in embryonic stem cells: Alu/SINE sequences tendentially associate with housekeeping genes, while LINE-1-enriched genes have specialized functions [83]. These data strongly suggest that retrotransposition events provide dynamic molecular ‘tools’ in the build-up of functional genomic circuits. While novel embryonic circuits are activated (C–E, C’–E’, F), the pre-existing ones (panels B, B’) are functionally inactivated but not erased; rather, they are taught to assume a non-functional, potentially reversible state that can be re-activated in response to appropriate stimuli. The model depicts the one- and two-cell stage embryonic transitions as the key window during which the sperm-delivered RNA-based information is decoded and drives the formation of novel genomic circuits that reshape the epigenetic landscape in the embryo, which is then stably acquired or ‘assimilated’ in the genome of the four-cell embryos.

## Permissive Context of Early Embryos and the Roles of LINE-1-Encoded RT

The sperm-delivered regulatory RNAs, albeit having low abundance, can effectively reshape the embryonic epigenetic landscape because zygotes and two-cell embryos represent highly responsive and variation-prone contexts. A key aspect in this process is the transient global abrogation of miRNA-dependent control in preimplantation embryos [84], which is thought to cause an increase of the stochastic fluctuations in gene transcription and generate conditions of high genomic instability [85, 86]. Consistent with this view, the nuclear organization of early embryos in different species is mostly unstructured before zygotic activation (at two-cell stage in mouse) and architectural features such as nuclear compartments, topological associated domains and loops are not yet established ([87], reviewed in Ref. [88] and references herein). By the eight-cell stage, chromatin compartments are fully established, similar to LADs. It is reasonable to hypothesize that during the unstructured phase, sperm-delivered regulatory RNAs can access the ‘open’ chromatin domains and begin reshaping the canalization process. This will reduce random fluctuations in the expression of target genes while concomitantly rewiring and stabilizing their transcription profiles [89].

In this framework, LINE-1 retroelements play another important role in addition to that associated with targeted retrotranspositions. LINE-1 expression is activated in preimplantation embryos [90, 91] and it is strictly required for early embryonic development, as LINE-1 transcription down-regulation, via antisense oligonucleotide injection, arrests developmental progression at the two-cell stage [92]. In addition, LINE-1 repression before the two-cell stage also results in decreased chromatin accessibility to DNase I concomitant with the decline in the developmental rate; in contrast, prolonged LINE-1 transcription prevents chromatin condensation [93], a naturally occurring phenomenon during developmental progression [94]. These results converge to indicate that high LINE-1 expression actually contributes to maintain an open chromatin conformation, a condition that facilitates the remodeling process in the early preimplantation stages.

**Figure 2:**
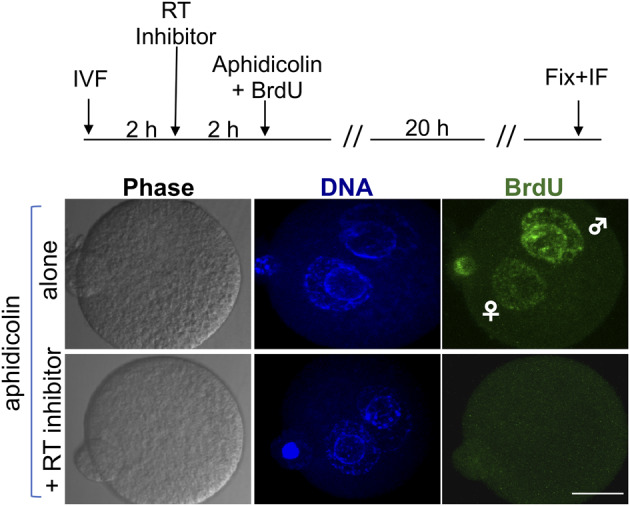
Reverse transcriptase-dependent BrdU incorporation occurs in zygote pronuclei. Upper panel: schematic representation of the protocol used to assess BrdU incorporation in parental pronuclei of in vitro fertilized (IVF) embryos in the presence of a DNA replication inhibitor (aphidicolin) or an RT inhibitor (abacavir). The immunofluorescence panels below show that BrdU incorporation (green) takes place in both parental pronuclei (blue), more intensely in the paternal one, independent on DNA replication, but is full inhibited in embryos exposed to the combination of aphidicolin + abacavir, indicating that is RT-dependent. Bar, 20 µm From Ref. [96].

LINE-1 retroelements encode their RT enzyme, that is active in preimplantation embryos [95, 96] and in epididymal spermatozoa ([42, 44, 45], reviewed in Ref. [41]). Earlier work showed that mouse zygotes incorporate 5^’^-bromodeoxyuridine (BrdU) cultured even in the presence of aphidicolin, a strong inhibitor of DNA replication, but this incorporation is abolished in the presence of the nucleoside RT inhibitor abacavir [96] which inhibits LINE-1-encoded RT [97]: thus, reverse transcription is ongoing in both paternal and maternal pronuclei (more pronouncedly in the former, see Fig. 2), suggesting a role in the targeted retrotransposition events (see above). The concomitance of RT activity with the abrogation of miRNA-dependent control in preimplantation embryos [77] suggests a possible link between the two processes.

Unrelated evidence comes from studies in cancer cells, which, similar to embryos, are also endowed with high levels of LINE-1-derived RT activity [98, 99]. Elevated RT abundance impairs the process of miRNA biosynthesis: indeed, abundant RT activity is associated with the formation of RNA: DNA hybrid molecules, generated via reverse transcription of miRNA precursors in cDNAs; this impairs the formation of double-stranded RNAs serving as substrates for Dicer cleavage into regulatory miRNAs RT activity inhibitors restore the normal miRNA transcriptome. In other words, the LINE-1-derived RT governs the balance between single- (or hybrid) and double-stranded RNA production. In early embryos, in which RT levels are high, RNA: DNA hybrids may similarly form, entailing the inhibition of miRNA biogenesis and the loss of miRNA-mediated control of gene expression. These conditions would favor stochastic variations in gene transcription [85, 86, 89] and contribute to keep early embryos in a permissive, variation-prone state with an unstructured chromatin condition.

### Assimilation of Epigenetic Information and Maintenance across Multiple Generations

Evidence at this point suggest roles of LINE-1-encoded RT in retrotransposition events and in the inhibition of miRNA-dependent control in early embryos. A third role is also emerging in replication and maintenance of the sperm-delivered RNA information.

Unlike lower organisms, mammals lack an RNA-dependent RNA polymerase for RNA replication. That function can be replaced by the interplay between RT and DNA-dependent RNA polymerase activities in a two-step process: RT first reverse-transcribes RNA molecules in cDNA copies, which are then transcribed back in multiple RNA copies. Mimicking a sort of ‘natural’ RT-PCR/PCR amplification cycle, this process contributes to amplify the RNA-based information in high copy numbers. Sperm cells are endowed with both RT and RNA polymerase activities [100] and can host such a process. That was shown in in vitro fertilization assays using epididymal spermatozoa that were pre-incubated with either β-gal or EGFP-encoding RNA and DNA molecules: the retro-transcribed/transcribed molecules were mosaic propagated as extrachromosomal sequences throughout embryogenesis and, later, in tissues of adult individuals, where they induced phenotypic variations [43, 44]. The reporter genes were further expressed in the next generation obtained from founders. Extrachromosomal information is thus generated in an RT-dependent manner, phenotypically expressed and gamete-transmitted to the next generation(s). The expression of such sequences may be limited to a few generations due to the persistence of the exogenous molecules rather than their stable integration. The model however predicts that a transient condition may be converted into a stable one and the newly formed regulatory genomic circuits may be permanently acquired. The ‘quantum’ of extrachromosomal RNA-based information delivered at each fertilization can be stabilized via the RT/RNA polymerase interplay and can progressively increase the load of sperm RNA storage accumulating across generations. It may be thought that, when the accumulation reaches above a critical threshold, it triggers the remodeling of the embryonic chromatin, contributing to the formation of novel stable genomic regulatory circuits with the emergence of permanent phenotypic changes. As recalled above, the RNA storage in spermatozoa is heterogeneous and susceptible to be influenced by external stimuli including various types of stressors. Ultimately, therefore, the ‘assimilation’, or permanent acquisition, of a novel epigenetic trait would depend on the nature, intensity and duration of the environmental stimuli experienced by the father. On the whole, the acquisition of novel regulatory circuits may be viewed as the final outcome of a process during which parental epigenetic information is converted into genomic novelty that can be stably acquired in the progeny. The ‘on-off’ RNA-driven remodulation of embryonic gene expression and the functional/structural contribution of retroelements indicate that the possible source of genomic pathways encoding altered traits in early embryos emerges from the interplay between epigenome and genome. This proposed model may also have strong implications for evolutionary processes.

## Conclusion

The exponential growth of epigenetic studies in recent years has evoked Lamarckism from its historical and scientific dismissal and has resumed Pangenesis to some extent, which was a rejected aspect of Darwinism [101, 102]. Both have strongly inspired the present model of transgenerational inheritance. The analogies are obvious: in the stream of information that flows from parental somatic tissues to the offspring, ECVs are the ‘updated’ molecular version of ‘gemmules’, the particles that, according to Darwin, mediate that flow; epididymal spermatozoa are their collectors and delivering vectors to oocytes. Triggers activating this amazing ‘assembly line’ are often provided by stressing stimuli and the final outcome is the acquisition of altered characters, supporting the assumption that somatically derived, extrachromosomal information can affect the embryo ontogenesis and generate permanently acquired variations/traits that become transmissible in a germline-dependent fashion. It is worth stressing that these events are possible because early embryos provide a molecularly ‘permissive’ environment due to the transient abrogation of the endogenous miRNA-dependent control [77]. Under these conditions, in the absence of endogenous Dicer-dependent miRNAs, the inherited sperm-delivered regulatory RNAs, depositary of parental epigenetic information, can unfold pronounced effects that reshape the expression landscape and generate variations that will affect the fate of the developing embryos.

On the one hand, this highlights the striking potential of living organisms to collect information and evolve accordingly, generating non-random genomic and phenotypic novelties; on the other hand, it must be pointed out that, at the same time, the remarkable stability of organismal genomes is even more striking. In order to maintain their stability in a continuously changing environment the genome must undergo continuous small adjustments that keep substantially invariant their global state, i.e. they reside in a ‘stable critical state’. In conclusion, genomes are complex networks self-controlling their global expression fluctuations [103], yet at the same time they are sensitive to myriads of stimuli, both environmental or internally programmed, that can perturb such stability. In these perspectives the fate of the organisms is determined by the balance between their buffering capacity and the potential of the stressing stimuli to overcome it.
